# Perspectives from the 2^nd^ International Post-Tuberculosis Symposium: mobilising advocacy and research for improved outcomes

**DOI:** 10.5588/ijtldopen.23.0619

**Published:** 2024-03-01

**Authors:** B.W. Allwood, R. Nightingale, G. Agbota, S. Auld, G.P. Bisson, A. Byrne, R. Dunn, D. Evans, G. Hoddinott, G. Günther, Z. Islam, J.C. Johnston, G. Kalyatanda, C. Khosa, S. Marais, G. Makanda, O.M. Mashedi, J. Meghji, C. Mitnick, C. Mulder, E. Nkereuwem, O. Nkereuwem, O.B. Ozoh, A. Rachow, K. Romanowski, J.A. Seddon, I. Schoeman, F. Thienemann, N.F. Walker, D.T. Wademan, R. Wallis, M.M. van der Zalm

**Affiliations:** ^1^ Division of Pulmonology, Department of Medicine, Faculty of Medicine, Health Sciences, Stellenbosch University and Tygerberg Hospital, Cape Town, South Africa;; ^2^ Department of Clinical Sciences, Liverpool School of Tropical Medicine, Liverpool,; ^3^ Department of Respiratory Medicine, Liverpool University Hospitals NHS foundation Trust, Liverpool, UK;; ^4^ University of Montpellier, Recherches Translationnelles sur le VIH et les Maladies Infectieuses (TransVIHMI), Institut de recherche pour le Developpement (IRD), Institut National de la Sante et de la Recherche Médicale (INSERM), Montpellier, France;; ^5^ Institut de Recherche Cliniques du Benin (IRCB), Abomey-Calavi, Benin;; ^6^ Departments of Medicine, Epidemiology, and Global Health, Emory University School of Medicine and Rollins School of Public Health, Atlanta, GA,; ^7^ Departments of Medicine and Biostatistics, Epidemiology, and Informatics, Division of Infectious Diseases and Epidemiology, Perelman School of Medicine at the University of Pennsylvania, Philadelphia, PA, USA;; ^8^ Thoracic Medicine Division of the Heart Lung Stream of St Vincent’s Hospital Sydney Faculty of Medicine, University of New South Wales, Sydney, NSW, Australia;; ^9^ Socios En Salud Sucursal, Lima, Peru;; ^10^ Division of Orthopaedic Surgery, Groote Schuur Hospital, Cape Town,; ^11^ Health Economics and Epidemiology Research Office, Faculty of Health Sciences, University of the Witwatersrand, Johannesburg,; ^12^ Desmond Tutu TB Centre, Department of Paediatrics and Child Health, Faculty of Medicine and Health Sciences, Stellenbosch University, Cape Town, South Africa;; ^13^ School of Public Health, Faculty of Medicine and Health, The University of Sydney, Sydney, NSW, Australia;; ^14^ Department of Pulmonary Medicine and Allergology, Inselspital, Bern University Hospital, University of Bern, Switzerland;; ^15^ Alliance for Public Health, Kyiv, Ukraine;; ^16^ University of British Columbia, Vancouver, BC, Canada;; ^17^ Division of Infectious Disease and Global Medicine, Department of Medicine, University of Florida College of Medicine, Gainesville, FL, USA;; ^18^ Instituto Nacional de Saúde, Marracuene, Mozambique;; ^19^ Division of Neurology, Department of Medicine, University of Cape Town & Neurology Research Group, Neuroscience Institute, University of Cape Town, Cape Town,; ^20^ TB Proof, Cape Town, South Africa;; ^21^ Kenya Medical Research Institute, Centre for Respiratory Diseases Research, Nairobi, Kenya;; ^22^ National Heart & Lung Institute, Imperial College London, London, UK;; ^23^ Program in Infectious Disease and Social Change, Harvard Medical School, Boston, MA, USA;; ^24^ Division TB Elimination and Health System Innovations, KNCV Tuberculosis Foundation, The Hague, The Netherlands;; ^25^ Department of Global Health, Amsterdam Institute for Global Health and Development, Amsterdam University Medical Centres, Amsterdam, The Netherlands;; ^26^ Vaccines and Immunity Theme, MRC (Medical Research Council) Unit The Gambia at The London School of Hygiene & Tropical Medicine, Atlantic Boulevard, Fajara, The Gambia;; ^27^ Department of Medicine, College of Medicine, University of Lagos, Lagos, Nigeria;; ^28^ Division of Infectious Diseases and Tropical Medicine, Medical Centre of the University of Munich (LMU), Munich, Germany;; ^29^ German Center for Infection Research (DZIF), Partner Site Munich, Germany;; ^30^ Unit Global Health, Helmholtz Zentrum München, German Research Center for Environmental Health (HMGU), Neuherberg, Germany;; ^31^ Department of Infectious Disease, Imperial College London, London, UK;; ^32^ General Medicine & Global Health (GMGH), Department of Medicine & Cape Heart Institute, University of Cape Town, South Africa;; ^33^ Department of Internal Medicine, University Hospital Zurich, University of Zurich, Zurich, Switzerland;; ^34^ Department of Medicine, University of Medicine and Dentistry of New Jersey, New Jersey Medical School, Newark, NJ, USA

**Keywords:** post-tuberculosis, Symposium, proceedings, advocacy, research

## Abstract

In 2020, it was estimated that there were 155 million survivors of TB alive, all at risk of possible post TB disability. The 2^nd^ International Post-Tuberculosis Symposium (Stellenbosch, South Africa) was held to increase global awareness and empower TB-affected communities to play an active role in driving the agenda. We aimed to update knowledge on post-TB life and illness, identify research priorities, build research collaborations and highlight the need to embed lung health outcomes in clinical TB trials and programmatic TB care services. The symposium was a multidisciplinary meeting that included clinicians, researchers, TB survivors, funders and policy makers. Ten academic working groups set their own goals and covered the following thematic areas: 1) patient engagement and perspectives; 2) epidemiology and modelling; 3) pathogenesis of post-TB sequelae; 4) post-TB lung disease; 5) cardiovascular and pulmonary vascular complications; 6) neuromuscular & skeletal complications; 7) paediatric complications; 8) economic-social and psychological (ESP) consequences; 9) prevention, treatment and management; 10) advocacy, policy and stakeholder engagement. The working groups provided important updates for their respective fields, highlighted research priorities, and made progress towards the standardisation and alignment of post-TB outcomes and definitions.

## RÉSUMÉ

En 2020, il est estimé qu'il y a 155 millions de survivants de la TB dans le monde, tous exposés à un risque d'invalidité post-TB. Le deuxième Symposium International Post-Tuberculose (Stellenbosch, Afrique du Sud) a été organisé dans le but de sensibiliser davantage à l'échelle mondiale et de permettre aux communautés touchées par la TB de contribuer activement à la mise en œuvre de l'agenda. De plus, nous avons entrepris de mettre à jour les connaissances sur la vie et les maladies post-TB, de déterminer les domaines de recherche prioritaires, d'établir des partenariats de recherche et de souligner l'importance d'intégrer les résultats sur la santé pulmonaire dans les essais cliniques et les services de soins de la TB. Le symposium était une réunion de travail pluridisciplinaire rassemblant des praticiens, des chercheurs, des personnes ayant survécu à la TB, des donateurs, des décideurs politiques et d'autres acteurs clés. Dix groupes de travail académiques ont établi leurs propres objectifs et ont abordé les sujets thématiques suivants : 1) engagement et perspectives des patients ; 2) épidémiologie et modélisation ; 3) pathogénie des séquelles post-TB ; 4) maladie pulmonaire post-TB (PTLD, pour l’anglais «*post-TB lung disease* ») ; 5) complications cardiovasculaires et vasculaires pulmonaires ; 6) complications neuromusculaires et squelettiques ; 7) complications pédiatriques ; 8) conséquences économiques, sociales et psychologiques (ESP, pour l’anglais «*economic-social and psychological*») ; 9) prévention, traitement et gestion ; 10) plaidoyer, politique et engagement des parties prenantes. Les groupes de travail académiques ont apporté des mises à jour significatives dans leurs domaines respectifs, ont mis en évidence les priorités de recherche et ont avancé vers la normalisation et l'harmonisation des résultats et des définitions de la post-TB.

It is estimated that 155 million survivors of TB were alive in 2020,^1^ for whom almost as much disability (as estimated by disability-adjusted life-years) will occur after ‘successful’ treatment, as during the disease itself.^2^ The 2^nd^ International Post-TB Symposium, held in Stellenbosch, South Africa, 17–19 April 2023, built on the work of the inaugural symposium in 2019,^3^ and was only the second gathering dedicated to the long-term consequences of TB and its multi-dimensional sequelae. The conference was organised by a 12-member Steering Committee from eight countries, composed of clinicians, researchers, TB survivors (as patient advocates), funders and policy makers. There were 152 delegates with 25 different occupations, representing 31 countries and 99 institutions. The aims of the symposium were 1) Advocacy: to increase global awareness of post-TB sequelae, and to empower TB-affected communities to play an active role in driving a person-focused TB agenda; 2) knowledge: to update knowledge on post-TB life and illness, and to identify research priorities; 3) networking: to build research collaborations, setting concrete plans for research and advocacy; and 4) consensus: to highlight the need to embed lung health outcomes in clinical TB trials and programmatic TB care services.

The symposium was divided into 10 thematic areas, with both plenary lectures and workshops allocated for all areas. These themes were 1) patient engagement and perspectives; 2) epidemiology and modelling; 3) pathogenesis of post-TB sequelae; 4) post-TB lung disease (PTLD); 5) cardiovascular and pulmonary vascular complications; 6) neuromuscular and skeletal complications; 7) paediatric complications; 8) economic-social and psychological (ESP) consequences; 9) prevention, treatment and management; and 10) advocacy, policy and stakeholder engagement. Each working group was chaired by a minimum of two people, with more than 100 individuals contributing to the preparation of the Symposium. Here, we present a summary of the proceedings, with further publications to follow from the individual working groups. Notable highlights included the inclusion of two new thematic areas—cardiovascular and pulmonary vascular complications, as well as neuromuscular and skeletal complications. In addition, new and revised definitions relevant to children for PTLD and post-TB meningitis were proposed. Consensus was achieved (using the Delphi process) on the need to develop a new research definition of PTLD, which will favour specificity for research purposes, and allow for between population comparisons. The construction and composition of this definition continues after the symposium using a Delphi process.

As part of the symposium’s goals, the Steering Committee highlighted the urgent need for inclusion of lung health outcomes (including standardised measures and tools) as part of current and future TB treatment trials. This is essential to determine the potential impact of novel TB regimens on disability outcomes and not merely microbiological endpoints.^4^ Further details on the Symposium, as well as video content of the presentations is available at www.post-tuberculosis.com, and Table 1 summarises important recommendations and research priorities.

**Table 1.** Summary of research priorities and recommendations from the working groups of the 2^nd^ International Post-TB Symposium, Stellenbosch, South Africa, 2023.

	Recommendations and research priorities
Patient engagement	Recommendation: Provide health information to people affected by TB at every step along the care cascade, including the need for follow-up after treatment completion Create safe spaces with ongoing counselling for families and communities affected by TB Use TB survivors’ narratives and mass media to unlock the power of advocacy for greater awareness and to facilitate action at community and policy levels
Epidemiology	Recommendation: For research purposes the “post-TB period” should be defined as beginning at treatment completion Prospective studies should monitor for post-TB outcomes for a minimum of 2 years post-TB treatment completion Studies collect at a minimum information on prior TB history; presence or absence of microbiologic confirmation of TB (e.g., using AFB smear, PCR or culture) and TB drug resistance; HIV status, treatment, CD4 count and viral suppression status; BMI and other measures of malnutrition; smoking history; and diabetes. Supplementary variables can include alcohol use, occupational exposure and mental health measures
	Research priorities: Defining minimum dataset requirements for prospective post-TB studies Risk stratification to understand who is most at risk for poor post-TB outcomes and the drivers of this accelerated decline Quantifying the burden of post-TB outcomes, including PTLD, post-TB cardiovascular disease and catastrophic costs
Pathogenesis and risk factors	Research priorities: Development of PTLD-specific representative animal models Identification and assessment of the importance of clinical risk factors for PTLD Identification of molecular and/or radiographic biomarkers as surrogate endpoints in clinical trials
Post-TB lung disease	Research priorities: Development of screening tools for the identification of TB survivors with clinically relevant PTLD at TB treatment completion Development of a robust diagnostic pathway for PTLD, which includes both recurrent TB and different PTLD-related conditions and subtypes, as well as a close-to-patients (accessible and affordable) diagnostic tools, including biomarkers Development of a more specific definition for research purposes, which includes both severity and certainty grades Development of health systems that can deliver equitable post-TB care, under the auspices of national TB programmes, but with strong links to non-communicable disease services
Cardiovascular and pulmonary-vascular complications	Research priorities: Research informing the risk and mechanisms of incident cardiovascular disease after TB Epidemiological data on excess mortality due to cardiovascular disease after TB Better epidemiological and outcomes data in post-TB pulmonary hypertension Development of methods for screening and confirmation of post-TB pulmonary hypertension, given limited global access to echocardiography and right heart catheterisation
CNS and musculoskeletal system	Research priorities: Determine the nature, frequency, and severity of disability in patients with CNS and musculoskeletal TB through standardised methods in prospective studies Identification of risk factors for neurology sequelae in patients with CNS and musculoskeletal TB Evaluate early intervention and targeted rehabilitation strategies in patients with CNS and musculoskeletal TB
Paediatric post-TB health and wellbeing	Recommendation: Children and adolescents should be included in post-TB disease studies
	Research priorities: Determine the proportion of children and adolescents who develop post-TB disease after treatment for pulmonary TB, TBM or spinal TB Determine the risk factors for post-TB disease in children and adolescents Determine the impact of post-TB disease on HRQoL of children and adolescents
Prevention, treatment and management of the Post-TB Lung Disease Working Group	Research priorities: Formal validation studies of a new instrument for assessing PTLD outcomes, noting the importance of performing them across multiple settings and populations Exploring host-directed therapies during and after TB therapy to modulate immune responses, with a view to prevent progressive lung damage Trials of therapies in established PTLD to improve HRQoL and long-term outcomes
ESP outcomes	Recommendation: Every person who completes treatment for TB should be evaluated for ESP outcomes alongside other sequelae at treatment completion and 1-year post-TB
	Research priorities: Increase primary data using standardised quantitative and qualitative measures on post-TB ESP outcomes, including across multiple high-burden settings, ages (including children and adolescents) and priority populations. Research further clarifying intersections, interactions, compounding and pathways between post-TB ESP-associated domains (Table 3) Intervention development, including studies to understand survivor priorities/preferences with effectiveness evaluations of interventions to 1) prevent and mitigate post-TB ESP sequelae, and 2) capitalise on TB survivorship
Advocacy	Recommendations: The inclusion of post-TB disease as an integral part of the natural progression of TB, advocating for its incorporation into national TB guidelines as an essential component of comprehensive care Active engagement with the media to launch impactful awareness campaigns Increase awareness of the extrapulmonary post-TB disease and disabilities Establishment of consortia, allowing various groups to work together and develop protocols tailored to the unique needs of each region

AFB = acid-fast bacilli; PCR = polymerase chain reaction; BMI = body mass index; PTLD = post-TB lung disease; CNS = central nervous system; TBM = tuberculous meningitis; HRQoL = health-related quality of life; ESP = economic, social and psychological.

## 1. PATIENT ENGAGEMENT WORKING GROUP

One of the aims of the Symposium was to place TB survivors’ voices at the centre of the post-TB community, and two TB survivors served as part of the Symposium Steering Committee. TB survivors also received a fee-waiver to attend, and time was allocated for multiple TB survivor stories to be shared during plenary sessions. The survivors’ gave moving accounts of their TB diagnoses, treatment and post-TB journeys, in particular, reflecting on challenges in accessing healthcare and how poor health systems response may have impacted their outcomes. Difficulties for TB patients and survivors in the context of their families and providing childcare was emphasised, and there was a word of appreciation for the increased recognition of the debilitating effect of post-TB-related disabilities, but calls for more to be done.

Person-centred care was the anchor around which this working group was organised, and the delegates explored ways in which they could prioritise person-centredness and engage patients. The importance of sharing health information with people affected by TB in appropriate ways at each step of the care cascade, including after treatment completion/cure, was discussed. Creating safe spaces with ongoing counselling for families and communities affected by TB was noted as an important step towards ongoing care. All delegates acknowledged the power of advocacy – especially through hearing TB survivors’ narratives – to generate awareness and facilitate action at both community and policy levels.

## 2. EPIDEMIOLOGY AND MODELLING WORKING GROUP

During the session, data on post-TB respiratory impairment, health-related quality of life (HRQoL) and post-TB health service use were presented.^5–7^ Presentations highlighted that people experience high rates of respiratory impairment, lower HRQoL and elevated healthcare use after successful treatment for TB disease. Challenges related to post-TB data were also highlighted. First, as the timing of post-TB outcome reporting varies,^5^ assessing trends between studies is challenging. Next, comparisons between sites remain difficult as groups use many tools to measure post-TB outcomes, particularly for post-TB HRQoL. Third, people who survive TB often have multiple pre-existing conditions and risk factors.^6^ As a result, determining how much of the adverse post-TB outcomes are due to TB vs. existing conditions remains challenging.

The workshop that followed was designed to explore and address these constraints. Our first objective was to develop a consensus on a standardised time for measuring post-TB outcomes. Discussions focused on when the post-TB period begins and, from a clinical and research perspective, what is a relevant post-TB period. Workshop participants agreed that TB-related damage likely starts at infection;^8^ however, from a pragmatic research perspective, we suggest the post-TB period begins once a successful treatment result has been recorded. Additionally, although there was group consensus that the post-TB period can be lifelong, from a research perspective, we suggest that ideally, prospective studies monitor for post-TB outcomes for a minimum of 2 years post-TB treatment completion. Implementing this timeline in future studies should result in more conclusive evidence to inform recommendations regarding what post-TB sequelae national TB programmes should monitor and for how long. Our second objective was to develop a consensus on a core set of pre-existing conditions and risk factors that may influence disease outcomes. We determined that while the core set of factors depends partially on the population, outcome of interest and data availability, we suggest studies collect at minimum, the following conditions 1) prior TB history 2) HIV, antiretroviral therapy (ART) and viral suppression status, 3) malnutrition and body mass index (BMI), 4) smoking history, and 5) diabetes. Additional variables of interest include alcohol use, occupational exposure and mental health measures. While standardisation of approaches to measuring these factors would be preferable, this is limited by varied access to resources between settings. There was also broad agreement that screening for many of these factors is insufficient. At minimum, screening must be combined with referral per standard of care in each setting. Finally, workshop participants were asked to specify their priorities for future research. Responses included 1) defining minimum dataset requirements for prospective post-TB studies; 2) risk stratification to understand who is most at risk for poor post-TB outcomes and the drivers of this accelerated decline; 3) quantifying the burden of post-TB outcomes, including PTLD, post-TB cardiovascular disease and catastrophic costs; and 4) the impact of risk factor intervention, such as smoking cessation programmes, on post-TB outcomes, including cost and HRQoL.

## 3. PATHOGENESIS AND RISK FACTORS WORKING GROUP

**Figure 1.**
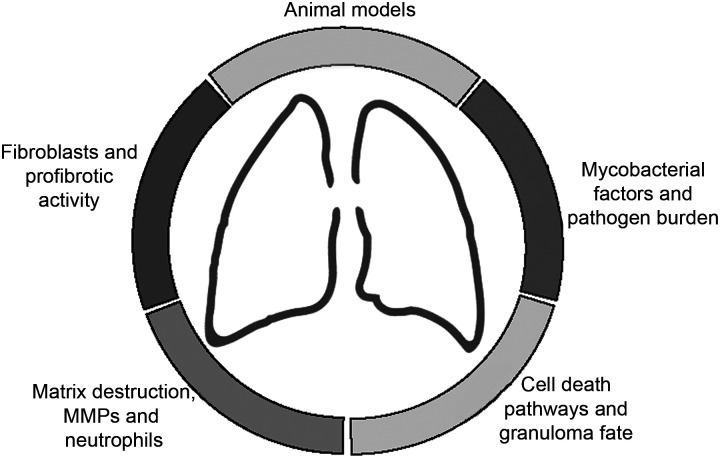
Focus areas in the pathogenesis of post-TB sequelae. MMP = matrix metalloproteinase.

This group aimed to identify key priorities for knowledge advancement around biological pathways and mechanisms important to post-TB pathogenesis, inform future research directions and generate novel hypotheses. In preparation for the workshop, the committee, (comprised of 13 experts with a range of clinical and scientific expertise), first established a focus on the pathogenesis of post-TB lung disease. Thereafter, the committee undertook a series of discussions and internal polls to identify six key areas for a focused review of the literature (see Figure 1). These were matrix destruction, including the role of matrix metalloproteinase dysregulation and neutrophil activity; fibroblasts and profibrotic pathways; cell death pathways and granuloma fate; mycobacterial factors, including pathogen burden; animal models; and the impact of clinical risk factors, including HIV, diabetes, smoking, malnutrition and alcohol*.* The plenary talk, informed by these reviews, summarised the state of current knowledge in each of these areas, setting the scene for the workshop and highlighting the gaps for discussion. The workshop was structured into breakout sessions to review key gaps and questions for the field and design a ‘dream’ study to address those gaps (Table 2). Research priorities identified include the development of PTLD-specific representative animal models, studies to identify and assess the importance of risk factors for PTLD, and identification of molecular and/or radiographic biomarkers that could be used as surrogate endpoints in clinical trials.

**Table 2.** Post-TB Pathogenesis Workshop Discussion topics: key gaps and questions for the field.

Topic area	Discussion summary
1) Experimental models	
What characteristics are necessary for a representative PTLD model (e.g., inoculum, stage, impact of clinical risk factors, etc.)? 1 How well do current animal models recapitulate key features of human disease? 2 What are the gaps in the fidelity of current animal models that limit our translation of their findings to human disease? 3 How best to address those gaps? 4 To what extent are the acute processes directly related to chronic processes? How to understand acute inflammatory damage vs. post-TB inflammatory damage and the overlap therein? 5 What are the key endpoints that should be evaluated in different preclinical models? 6 What animal models can incorporate functional outcomes?	No existing studies on post-TB sequelae in animals Need to develop models as a first step, benchmark against tissue pathology and function in human samples to validate Ideally, evaluation of function in animals, and radiology e.g., spirometry and CT imaging
2) Importance of mycobacterial burden	
How to tease out the impact of mycobacterial burden, strain, antigenic stimulation? 1 Convergence of host and bacterial factors, how to best study/understand? 2 Rate/nature of clearance and pathogen killing/cell death and different PTLD outcomes? 3 How to anticipate impact of new TB treatment regimens on variable tissue outcomes?	Importance of sputum and blood samples at baseline for determination of association with later functional outcomes, e.g., o Strain/whole-genome sequencing of bacteria o Microbiome sampling Determining cause and effect with respect to bacterial burden vs. lung damage o Impact of cavitation, bronchiectasis Relationship with antigen burden (including with bacterial killing) and inflammation, tissue destruction Autopsy studies with forensic controls (healthy lungs)
3) Matrix destruction and remodelling	
How to design studies to understand temporal sequence of tissue damage and remodelling and how these impact the development PTLD? 1 Consider testing host-directed therapies and small molecule inhibitors	Importance of starting with early TB, to look at point of onset of tissue damage; PET-CT offers a modality to quantify/assess Importance of baseline timepoints when collecting samples longitudinally, key question is what should be considered the baseline of post-TB pathology Magnitude of initial inflammatory insult likely to be important; natural history is then fibrosis and scarring One approach is to consider available immunomodulatory pathway inhibitors (therapeutics) and then study those inhibitors in human experimental medicine studies/trials to better understand role of those pathways
4) Clinical evaluation/“dream study” conceptualisation	
How to design a clinical study to evaluate mechanism, and the impact of clinical risk factors?	Large prospective longitudinal studies, including non-TB and birth cohort studies to understand key risk factors Utilise existing biobanks effectively; importance of post-mortem/surgical samples Use of CT-PET to evaluate (subclinical) Inflammatory pathology Building onto RCTs, to assess impact of interventions vs. placebo Impact of TB-IRIS, HIV, diabetes, undernutrition, alcohol, smoking, TPT (as “natural” experiments) Pre-clinical studies required to inform clinical hypotheses; clinical studies to have exploratory laboratory components Combined endpoints including spirometry, radiology, echocardiography; importance of evaluating inflammatory markers, epigenetics as predictive biomarkers
1 Brainstorming on clinical study design, thinking about innovative study design, modelling, etc. 2 Figure on translational spectrum and iterative nature of discovery from bench to bedside and back 3 How/what/when to measure baseline clinical characteristics and samples to inform mechanistic insights that can be taken to preclinical studies? 4 How to embed mechanistic questions into clinical trials? 5 What are the key endpoints that should be evaluated in clinical studies? 6 Can we determine valid surrogate endpoints for PTLD that could be assessed earlier?	


PTLD = post-TB lung disease; CT = computed tomography; PET = positron emission tomography; RCT = randomised controlled trial; IRIS = immune reconstitution inflammatory syndrome; TPT = TB preventive therapy.

## 4. POST-TUBERCULOSIS LUNG DISEASE (PTLD) WORKING GROUP

**Figure 2.**
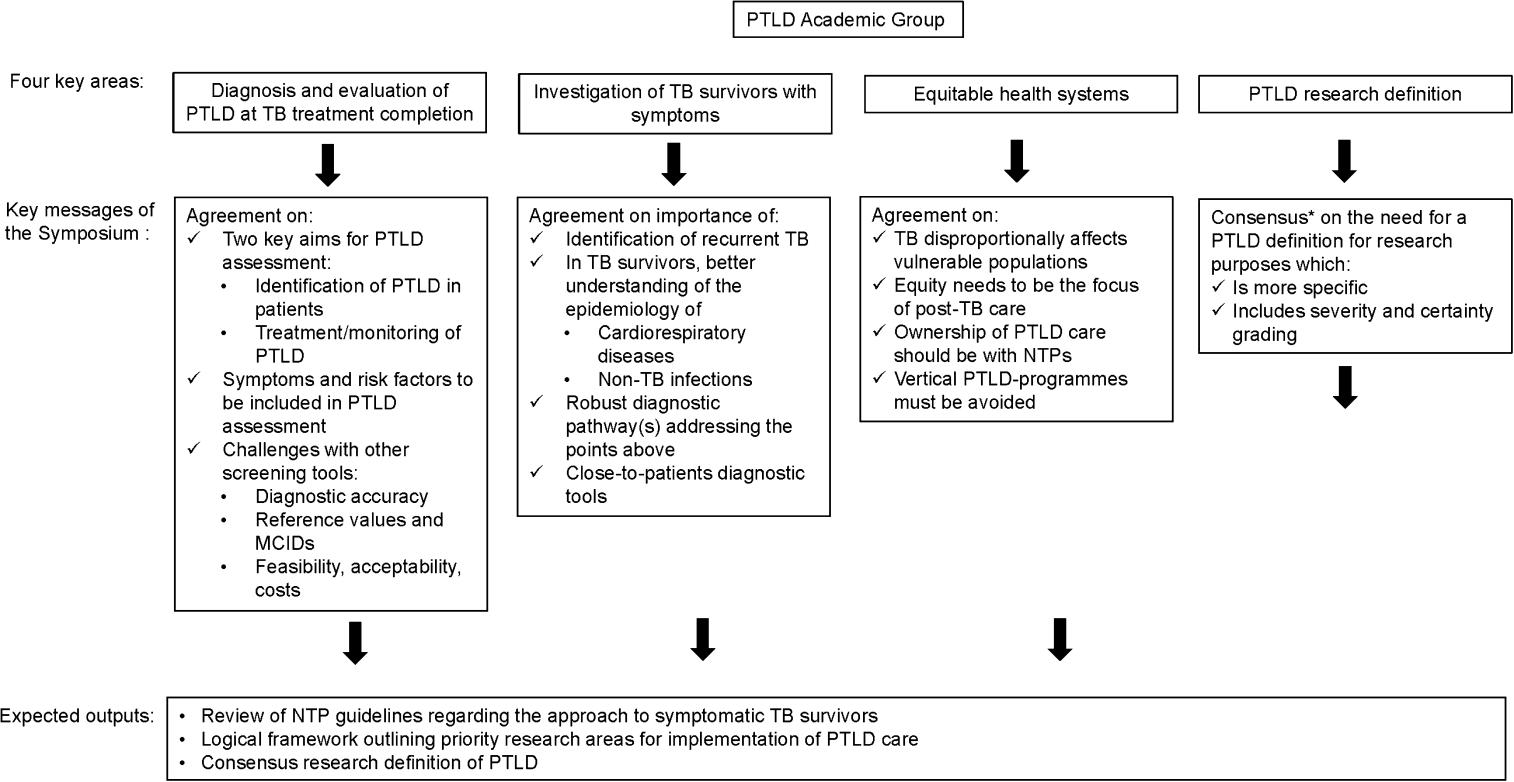
Summary of Proceedings from the Post-Tuberculosis Lung Disease (PTLD) Working Group. *Delphi voting. PTLD = post-TB lung disease; MCID = minimal clinically important differences; NTP = national TB programme.

PTLD has recently been included in the Global Plan to Stop TB, as well as in the Global Fund´s funding note for national TB programmes (NTPs).^9,10^ This offers an opportunity for programmes to address key issues around the design and implementation of post-TB care in high TB incidence settings.^3,11,12^ The working group focused on exploring four implementation questions considered of high priority for clinicians, health systems or the scientific community, with four working groups to explore these areas through discussion, evidence review and synthesis prior to the meeting, and through interaction with all symposium delegates within two workshops at the meeting itself (Figure 2). Key messages 1–4 resulting from this work are briefly outlined below, with outputs to follow, including a review of NTP guidelines to understand current approaches to the investigation and management of symptomatic TB survivors, a logic framework outlining priority research areas for the implementation of post-TB care and a consensus research definition of PTLD.

1. Screening for PTLD within health systems of high TB burden countries: the implementation of PTLD screening at the end of TB treatment was discussed with the aim of identifying TB survivors at risk for developing clinically relevant PTLD and related adverse health outcomes, and the identification of symptomatic patients for linkage to care. There was some uncertainty about whether screening should identify only symptomatic PTB survivors, or those without symptoms but with abnormal imaging or spirometry results. However, the majority of experts agreed that respiratory symptoms and risk factor screening should be part of any PTLD assessment, with lack of consensus on the role of chest X-ray, spirometry and physical capacity tests such as the 6-minute walk test. Perceived challenges with all screening tools include diagnostic accuracy in PTLD subgroups, methodological challenges around identifying reference values or minimal clinically important differences, and aspects of feasibility, acceptability and cost. We acknowledge that further data on which patients develop adverse outcomes over time will be needed to inform this, and that screening approaches may need to be a flexible response to the interventions available for patients and the inclusion criteria for these.
2. Clinical evaluation of TB survivors with residual or recurrent respiratory symptoms: although this patient group was well recognised, few NTP guidelines outline a diagnostic approach for symptomatic TB survivors returning to care. Where this is included, the need to identify recurrent TB is prioritised, and almost no guideline addresses broader investigation for non-TB respiratory infections or cardiovascular diseases. There was consensus around the need for a better understanding of the epidemiology of cardiorespiratory disease and non-TB infections among returning symptomatic TB survivors, a robust diagnostic pathway which includes both recurrent TB and non-TB, PTLD-related disease, and close-to-patients diagnostic tools for use in this pathway.
3. Preparedness of health systems to adopt PTLD care: TB disproportionally affects vulnerable populations who may face barriers in accessing care. There was broad agreement that a focus on equity is needed as pathways to post-TB care are developed. The WHO health system framework was used to explore aspects of service development, with specific focus on the governance of care, with agreement that NTPs may be best placed to take ownership of services with broader input from non-communicable disease (NCD) services, community services and broader civil society. There was agreement that vertical post-TB programmes must be avoided in this context.
4. Development of a research definition for PTLD: at the 1^st^ International Post-Tuberculosis Symposium, the delegates adopted the first consensus definition of PTLD as, ‘Evidence of chronic respiratory abnormality, with or without symptoms, attributable at least in part to previous tuberculosis’.^3^ This definition is sensitive, inclusive, flexible and useful for patient care and advocacy. However, delegates at the second symposium using a Delphi process (with majority consensus threshold set at 85% or greater), agreed that a more specific definition for PTLD is needed for research purposes, which includes both severity and certainty grades. Possible criteria for grading scores were discussed at the workshop and Delphi activities of voting towards a new definition are currently ongoing.

## 5. CARDIOVASCULAR AND PULMONARY VASCULAR DISEASE WORKING GROUP

This was a new thematic area and the working group focused on presenting the state of knowledge in this area. Cardiovascular disease (CVD) consequences of TB have been largely ignored. Although pericarditis remains a common manifestation of TB, extrapulmonary TB disease such as granulomatous arteritis are rare direct manifestations. It has become clear, however, that generalised immune activation may lead to CVD and there is protracted immune activation beginning before active TB, persisting years after TB is treated. Epidemiologic data on the association between TB and CVD are limited but compelling.^13–16^ These retrospective studies suggest an association between immune activation, TB infection and CVD, including acute myocardial infarction and coronary artery disease. However, data from prospective studies to understand causality and pathogenesis are lacking. Understanding of risk factors will aid development of primary and secondary interventions such as the role of antiplatelet therapy or statins.

Remarkably little is known about the development of pulmonary hypertension (PH) after TB, despite being documented in the literature over 70 years ago.^17^ Although TB was listed as a cause in 38.8% of PH associated with chronic lung disease in China,^18^ the incidence is unclear, but has been documented both during TB treatment (PH active-TB) and after treatment completion (PH post-TB). However, studies are biased towards hospitalised patients and patients with more severe disease. Furthermore, the relationship between PH active-TB and PH post-TB is unclear. Recent estimates for PH post-TB range from 67% in patients with respiratory failure, to 42% for hospitalised patients and symptomatic outpatients, and up to 6% of non-healthcare seeking outpatients.^19^ Early data show no associations with spirometry, HIV and 6-minute walk distance, but a possible association with cigarette smoking and multiple episodes of TB.^20^ The relationship between parenchymal damage and presence of PH is unclear, with univariate associations not maintaining significance after adjustment for confounders.^21–23^ However, PH post-TB appears to have higher mortality and hospital readmission rates than PH in other chronic lung diseases.^24^ The pathogenetic mechanism of PH post-TB remains to be understood, and potentially include parenchymal damage, primary arteriopathy, in-situ thrombosis or embolism, as well as fibrosing mediastinitis in a minority of persons with TB.^25,26^ The prevention and optimal treatment of PH post-TB is not yet known. Advanced PH therapies may potentially have detrimental effects, and focus is currently to optimise the underlying lung disease, including providing supplemental oxygen when indicated.^27^

Important research priority areas were identified. For CVD, these included the urgent need for better and preferably prospective data on the increased risk and mechanisms of incident CVD post-TB, as well as consensus around assessment for CVD outcomes for future research. With respect to post-TB PH, the classification of post-TB PH into Group 3 PH (secondary to chronic lung disease) or Group 5 PH (due to multifactorial mechanisms) is controversial and was discussed without conclusion. The urgent need for better epidemiological and outcomes data in post-TB PH was highlighted. However, this hinges on screening, and both the timing and methods of screening and confirmation for post-TB PH remains difficult in the majority of high-burden TB settings, where access to echocardiography and right heart catheterisation is limited. Ideally, high-risk populations need to be identified, to allow for targeted screening and better resource utilisation.

## 6. CENTRAL NERVOUS SYSTEM (CNS) AND MUSCULOSKELETAL SYSTEM WORKING GROUP

This was a new thematic area, and an overview of the current knowledge of neurological sequelae following central nervous system (CNS) TB was provided. TB meningitis (TBM) is frequently considered the most devastating form of TB, with one trial reporting death during treatment in 19% of HIV-negative and 39% of HIV-positive adults (*n* = 817),^28^ and 40% disability among survivors. Low cognitive performance after TBM, as measured by the global disability score, has been reported in up to 47% of HIV-associated TBM patients at 6 months follow-up.^29^ In children, one study (*n* = 327) found 22% of survivors had a poor outcome as defined by a developmental or intelligence quotient of <50 at the end of TBM treatment.^30^ Other forms of CNS TB are similarly associated with significant morbidity after treatment completion; for example, a retrospective review found 56% of adults with spinal TB (*n* = 87) were unable to walk after 9 months.^31^

A summary was also presented on disability, treatment and outcomes in patients with musculoskeletal TB, including the devastating physical impairment suffered by these largely paediatric patients, in terms of spinal deformity, paraplegia and peripheral joint destruction.^32,33^ The predominant anatomical area affected is the spine, with the hip and knee also commonly affected. The insidious onset of disease and vulnerable population affected (having poor access to appropriate care) were highlighted as a reason for the late presentation when pathology was advanced, with resultant long-term sequelae of joint destruction, impaired ambulation and pain. Surgical interventions were presented to indicate the magnitude and cost required to restore function. Discussions focused on the lack of knowledge around the extent of disability following treatment of neuromuscular and skeletal TB. Currently neurocognitive assessment following TBM is not standardised and detailed assessment is rarely performed due to time constraints and lack of validated culturally appropriate tools.^34,35^ Although other neurological sequelae of CNS and spinal TB such as seizures and motor, sensory and sphincter dysfunction are commonly encountered by clinician practitioners, their frequencies, severity and impact on patients’ HRQoL are rarely reported.^11^ Furthermore, evidence-based strategies to manage these complications are lacking. Patients with CNS and musculoskeletal TB are at increased risk of incurring catastrophic costs (i.e., spending at least 20% of annual household income on TB diagnosis and care) compared to patients with pulmonary TB, and these costs were discussed more fully in the ESP working group.

Increased awareness and evidence-based management of disability associated with CNS and musculoskeletal system TB are urgently required. It was concluded that future studies should include 1) assessment of the nature, frequency and severity of disability in patients with CNS and musculoskeletal system TB through standardised methods in prospective studies; 2) identification of risk factors for neurology sequelae in patients with CNS and musculoskeletal TB, including the influence of different antimicrobial and host-directed therapies on outcome; and 3) to evaluate early intervention and targeted rehabilitation strategies in patients with CNS and musculoskeletal TB to improve long-term outcomes.

## 7. PAEDIATRIC POST-TB HEALTH AND WELLBEING WORKING GROUP

Data have recently emerged regarding PTLD in children, indicating a correlation between pulmonary TB and subsequent respiratory morbidity, including lung impairment.^36,37^ Data from a birth cohort study showed that respiratory morbidity after TB was independent of premorbid lung function, indicating a direct effect of TB.^36^ The paediatric working group focused on two primary disease entities: PTLD and post-TBM disease. Within these areas, the group’s discussions centred on defining research definitions, developing a toolbox, establishing standardisation, including the assessment of HRQoL and outlining research priorities. The research definition from the 2019 symposium was reviewed and slightly modified, by replacing ‘adequately treated’ with ‘treated’^3^ to state: ‘Evidence of chronic respiratory impairment in an individual previously treated for pulmonary tuberculosis in whom active tuberculosis is excluded, and in whom no other cause of chronic lung disease is the predominant cause.’ The group felt it was not possible to be more specific. However, they decided, similar to the overall PTLD Delphi, to use the toolbox to add layers to the definition, describing the severity and type of impairment in more detail. The toolbox was discussed, taking inspiration from the toolbox developed and proposed for adult PTLD,^3^ but with the inclusion of additional assessment tools specific to children and adolescents. There was a strong interest in standardising and aligning lung health outcomes in children and adolescents, as well as providing comprehensive guidance on good quality assessment of lung health in this population. This will be done via an expert consensus statement and separate publication.

Post-TBM disease was also considered by the group, and an initial discussion was held regarding a research definition. In line with the research definition for PTLD, the group agreed upon the following definition: ‘Evidence of chronic neurological, cognitive, behavioural and developmental impairment in an individual previously treated for tuberculosis meningitis in whom active tuberculosis is excluded, and in whom no other cause of impairment is the predominant cause.’ Once again, the group recognised the potential for further classification by utilising a toolbox to define and standardise neurological outcomes.

The assessment of HRQoL was considered crucial and overlapping in evaluating post-TB health in all children and adolescents. Various quantitative tools for measuring HRQoL in this age group were considered.^38–41^ However, there is currently no disease-specific HRQoL tool, and most existing tools lack depth and an understanding of the socio-economic context specific to high TB burden settings. The working group unanimously agreed that a combination of qualitative and quantitative measures are necessary to assess post-TB HRQoL in children and adolescents comprehensively. Research priorities are reflected in Table 1.

## 8. PREVENTION, TREATMENT AND MANAGEMENT OF THE POST-TB LUNG DISEASE WORKING GROUP

**Figure 3.**
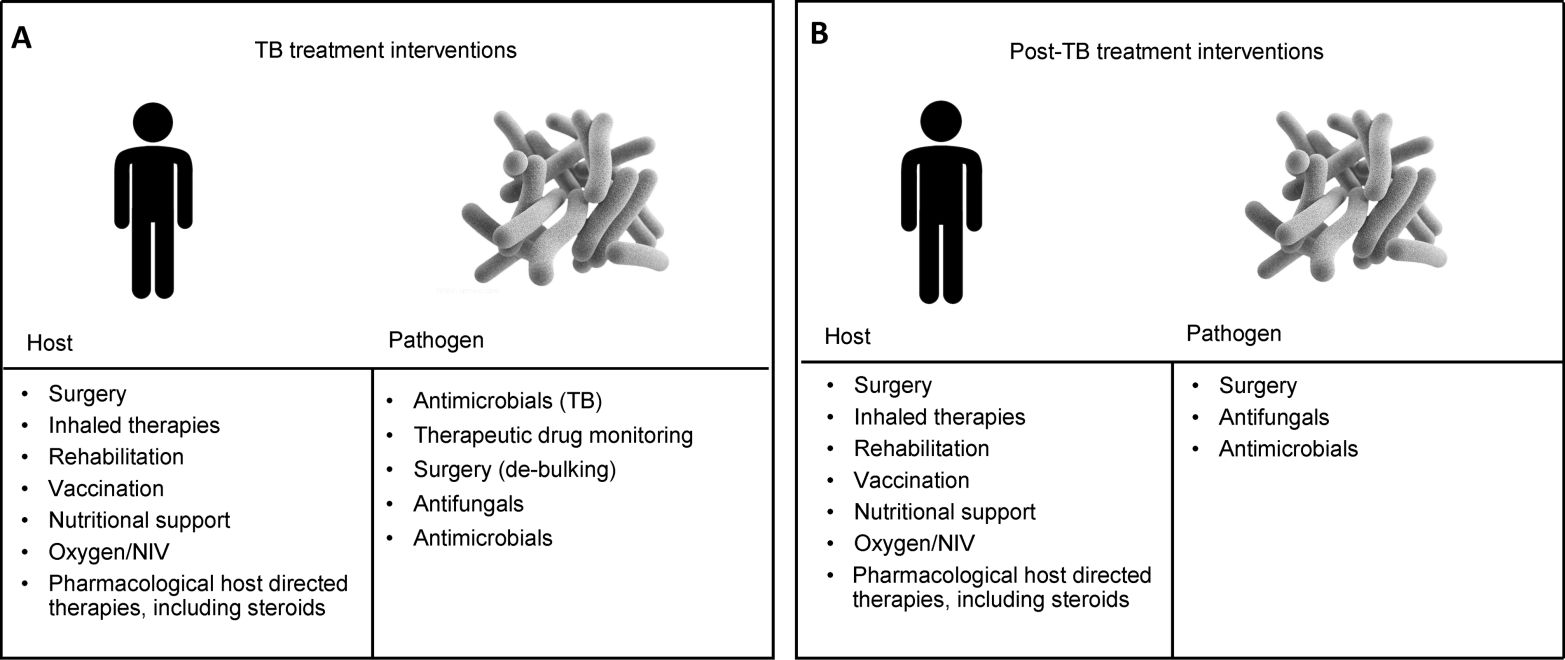
Conceptual framework of interventions on PTLD: **A)** target and timing of intervention; **B)** type of intervention by target and timing. PTLD = post-TB lung disease; NIV = non-invasive ventilation.

The evidence base and conceptual framework for the prevention, treatment and management of PTLD were explored. A wide range of interventions has been used to prevent and treat this heterogeneous condition. Published reports use a diversity of definitions of exposures and outcomes. For these reasons, the working group conducted a scoping, rather than systematic, literature review. We classified the studied interventions according to their target (pathogen or host) and timing (during or after TB treatment). The review and workshop discussion identified several potential interventions to prevent and manage PTLD. This conceptual framework and key interventions are shown in Figure 3. The detailed methods and findings of the scoping review are currently in preparation as a separate manuscript.

The decision to not combine outcomes reported across the diversity of studies also reflected the absence of a validated instrument to screen for PTLD and/or distinguish it from other respiratory conditions. PTLD signs and symptoms are non-specific and overlap with those of many chronic respiratory conditions of other aetiologies. Tools used to screen for these other pathologies may not perform well for PTLD. A purpose-built instrument to establish PTLD and to assess changes over time does not yet exist. Development of such an instrument is a priority for research in PTLD. It should be relatively short, well-performing and simple to use in the target populations. In a pilot Delphi process the workshop attendees supported the need to develop such an instrument and considered various other tools that might contribute to development of such a scale. The group also endorsed formal validation studies of a new instrument, noting the importance of performing them across multiple settings and populations. Possible platforms in which such validation studies could be performed include TB-SEQUEL, IeDEA (International epidemiology Databases to Evaluate AIDS) and other ongoing cohort studies as well as future trials. Results from the PURE (Prospective Urban Rural Epidemiology) study (among others) highlight the prognostic relevance of forced expiratory volume in 1 sec (FEV_1_) in lung function testing as a predictor for mortality, CVD and hospitalisation for respiratory causes.^42^ The role of lung function impairment alone in PTLD remains less clear,^43^ but evidence suggests that FEV_1_ and exacerbations of lung disease could be targeted with pharmacological interventions to address PTLD. Based on spirometry, FEV_1_ can be assessed in many settings, while more advanced diagnostics like lung volumes and diffusion capacity are less accessible. Despite all limitations of FEV_1_ in spirometry, it seems currently the best evaluated and most accessible parameter to describe lung function impairment secondary to PTLD on a population basis.

The potential exists for host-directed therapies during and after TB therapy to modulate immune responses, thereby preventing otherwise progressive structural lung damage.^44,45^ These disease-modifying drugs for TB (TB-DMDs) promise to preserve lung function post-TB much as DMDs have done for rheumatic diseases. Subsequent to the development of PTLD, vaccination against other respiratory pathogens may prevent additional morbidity,^46^ and pulmonary rehabilitation may prove to be a low-cost intervention that can be adapted to local conditions.^11,12,47^

## 9. ECONOMIC, SOCIAL AND PSYCHOLOGICAL OUTCOMES WORKING GROUP

Since the 1st Symposium,^3^ the Economic, Social, and Psychological (PTLD ESP) working group had grown from 5 to 33 attendees (with representatives from 17 countries) who participated in pre-, during and post-Symposium activities. This is indicative of increasing attention being paid to the well-being of TB survivors after completing TB treatment. Recent modelling studies,^2,48,49^ the Clinical Standards for PTLD^12^ and a consensus statement for post-TB health and well-being,^11^ emphasise the ESP impact of PTLD. Emerging data suggest that TB survivors who describe residual respiratory symptoms also report progressive deterioration in general and disease-specific HRQoL, ongoing psychological distress, poor exercise capacity and dissaving (i.e., spending in excess of their current earnings). Temporal trends data from observational studies have shown that outcomes are typically lowest/most severe at the start of TB treatment, with the greatest improvement observed during TB treatment, but with limited recovery in the year after treatment completion.^50^ However, empirical evidence (both qualitative and quantitative) remains sparse. As noted in the literature^11,51,52^ and other PLTD working groups, this group also noted a paucity of data on the long-term economic, social and psychological effects of TB on survivors generally, but especially children and adolescents. While more is known about the direct impact of TB on children and adolescents,^37,51–53^ less is known about the indirect impact. Some of these consequences include impoverishment, stigma, neglect/violence, family separation, effects on nutrition and food security and missed education opportunities, which can have lifelong consequences for those affected.

The group agreed that new study protocols must embed measurement of PTLD ESP correlates and outcomes, including establishing the feasibility of assessing PTLD ESP outcomes under routine programmatic conditions. To facilitate this, the working group focused on 1) identifying the domains relevant to PTLD ESP (Table 3), 2) considering intersections between underlying ESP vulnerabilities, experiences during the disease episode with both immediate and delayed latency, and ESP consequences of PTLD biological sequelae, and 3) formulating a working list of standardised PTLD ESP outcomes and measures (Table 4). The consensus is that every patient should be evaluated for these outcomes alongside physical disability at treatment completion and at a minimum at 1-year post-TB; however, additional follow-up is encouraged, particularly, if prospective studies are monitoring post-TB outcomes for a minimum of two years post-TB treatment completion.

**Table 3.** Responses elicited from the post-TB ESP working group on the most important features that could be considered for each domain.

Psychological domains	Social domains	Economic domains
o Anxiety and/or depression (10) o Self-stigma (8) o Mental health (4) o Hopelessness and doubtfulness (3) o Self-esteem and confidence (3) o Shame or guilt (2) o Substance use disorders (2) o Cognition/decision-making (2) o Growth and development (2)	o Changes to social networks (dynamics) and relationships (9) o Poor academic performance at school (5) o Stigma (experience) (5) o School attendance (interrupting or dropping out of school) (4) o Loss of relationships (3) o Social support (2) o Taking part in social activities and physical functioning) (2)	o Loss of income (10) o High costs to attend the clinic (5) o Reduced future earnings (4) o Ability to work (3) o Employment and economic recovery (3) o Food insecurity (2) o Continue to experience catastrophic costs post-TB (2)

ESP = economic, social and psychological.

**Table 4.** Commonly reported PTLD and post-ESP outcomes and the tools used to measure these.

Outcome	Tool	Outcome	Tool	Outcome	Tool
Health-related quality of life	Adults: SGRQ (disease specific) WHOQOL-BREF EUROHIS-QOL 8 (TB specific) QLICD-PT - pulmonary TB-specific QOL scale EuroQOL-5D MOS SF-36/SF-12	TB-related stigma	Van Rie's TB Stigma Scale Adjustments and additional items for the general worker population^54^ Social Adaptation Self-evaluation Scale (SASS) The Participation Scale (Van Brakel) Social Functioning Scale (SFS) Redwood self-stigma sub-scale	Psychological distress	Kessler Psychological Distress Scale (K10) Patient Health Questionnaire 9 (PHQ-9) Self-rating depression scale Beck's Depression Inventory Depression, Anxiety & Stress Scale (DASS 21) Hospital Anxiety Depression Stress scale (HADS) Center of Epidemiologic Studies Depression Scale, 10-item version (CES-D-10)
	Paediatrics: EQ-5D-Y and TANDI Generic PedsQL V.4.0				
Pain	Pain Impact Questionnaire (PIQ-6); Visual Analogue Scale (VAS)	Food security	Household Food Insecurity Access Scale (HFIAS) 24-hour food recall diary (dietary diversity)	Sleep	Sleep Measurement Scale Insomnia Severity Index Pittsburgh Sleep Quality Index Epworth Sleepiness Scale
Disability	Sheehan Disability Scale Severe Respiratory Insufficiency (SRI) Questionnaire BOLD core questionnaire Brief MDS disability questions	Employment recovery	WHO patient cost survey Hours worked Employment Work interruptions Income Household assets	Cognitive function (functional capacity)	Balance and gait Grip strength using a muscle tester Brief Balance Evaluation Systems Test (BESTest) Balance confidence Mini-Mental State Examination Berg Balance Scale (balance fall risk)

PTLD = post-TB lung disease; ESP = economic, social and psychological; SGRQ = St. George's Respiratory Questionnaire; WHOQOL-BREF = World Health Organization Quality of Life Brief Version; BOLD = Burden of Obstructive Lung Disease Initiative; MOS = Medical Outcomes Study; SF-36 = 36-Item Short Form Survey; TANDI = Performance of the Toddler and Infant; QLICD = Quality of Life Instruments for Chronic Diseases; PedsQL = Pediatric Quality of Life Inventory.

Three recommended priorities for future research are 1) increased primary data using standardised quantitative and qualitative measures on PTLD ESP outcomes, including across multiple high-burden settings, ages (including children and adolescents) and priority populations; 2) conceptual, multidisciplinary and mixed-method research further clarifying intersections, interactions, compounding and pathways between PTLD ESP-associated domains; 3) intervention development, including studies to understand survivor priorities/preferences and using human-centred and participatory co-design approaches; followed by 4) effectiveness evaluations of interventions i) to prevent and mitigate PTLD ESP sequelae, and ii) to capitalise on TB survivorship.

## 10. ADVOCACY, POLICY AND STAKEHOLDER ENGAGEMENT WORKING GROUP

The workshop addressed the current state of post-TB awareness and explored strategies to enhance it through advocacy, policy influence and stakeholder engagement. The workshop incorporated presentations and discussions delivered by representatives from international aid organisations, policymakers, non-governmental organisations (NGOs), community engagement specialists, individuals directly impacted by TB and TB survivors. During the discussions, participants acknowledged existing barriers and drew valuable insights from successful experiences in advocacy and policy change. Although delegates reported some parts of the world were aware of this condition, there was a notable lack of awareness in other high-incidence countries. Participants recognised the challenges in raising awareness and advocating for post-TB disease without a standardised definition and guidelines for diagnosis and treatment. The lack of funding for research to gather evidence, including proof of effectiveness and feasibility of interventions that could then inform policymakers, further exacerbated these difficulties. The workshop emphasised that TB has broader consequences beyond health, as it significantly impacts family life, social interactions, stigma and physical activity and can lead to economic hardships. The workshop proposed the following measures: 1) the inclusion of post-TB disease as an integral part of the natural progression of TB, advocating for its incorporation into national TB guidelines and treatment algorithms as an essential component of comprehensive care; 2) active engagement with the media to launch impactful campaigns focused on raising awareness and creating community-based demand for the treatment of this condition; 3) increased awareness about the non-pulmonary post-TB disease and the associated disabilities resulting from TB, aiming to ensure that these aspects receive adequate attention and understanding; and collaboration and knowledge sharing while addressing region-specific challenges, and the establishment of consortia, allowing various groups to work together and develop protocols tailored to the unique needs of each region.

The workshop recommended fostering engagement and collaboration with civil society groups, NGOs, healthcare workers, professional bodies, TB survivors and organisations involved in public–private partnerships. The objective is to collectively raise awareness about post-TB disease and work towards establishing a standardised research definition of the condition and developing protocols for its diagnosis and treatment.

## CONCLUSIONS

The 2^nd^ International Post-Tuberculosis Symposium stated objective was to increase advocacy and improve patient-centred outcomes for TB survivors. This was achieved by empowering TB survivors, placing them at the centre of the symposium. TB survivors featured in the plenary sessions with the inclusion of the voices and lived experiences of TB survivors. A clear statement was made by the steering committee for the urgent need to include lung health outcomes in all new clinical trials for TB treatment to allow important secondary outcome comparisons of respiratory impairment between the treatment regimens. Clinical trials provide an ideal opportunity to systematically collect clinically relevant information that has potentially important consequences for population (lung) health, when new treatment regimens are rolled out on a programmatic level.^4^

The academic working groups provided important updates for their respective fields and made progress towards the standardisation and alignment of post-TB outcome definitions, with the aim of improving collaborative research and allowing comparisons of research data across different settings. Finally, the symposium highlighted the importance of including the patient care cascade in the disease spectrum of TB that oscillates from exposure to ‘pre-TB’, active disease and post TB. The incorporation of TB-associated morbidity (post-TB) into National TB Guidelines is also an essential component of comprehensive, patient-centred TB care, as is the development of both international and regional guidelines (e.g., the recent recommendations in Brazil^47^). Through these steps from advocacy to action, the symposium Steering Committee, academic working groups and delegates aim to improve the long-term health outcomes and quality of life for all children, adolescents and adults that survive TB disease. The next symposium Steering Committee was elected during the symposium and will expand to ensure continued global representation of the post-TB community.
